# Effects of Serious Games for Patients With Chronic Obstructive Pulmonary Disease: Systematic Literature Review

**DOI:** 10.2196/46358

**Published:** 2023-09-25

**Authors:** Houqiang Huang, Min Huang, Qi Chen, Mark Hayter, Roger Watson

**Affiliations:** 1 Nursing Department The Affiliated Hospital of Southwest Medical University Luzhou China; 2 Department of Respiratory and Critical Care Medicine The Affiliated Hospital of Southwest Medical University Luzhou China; 3 Department of Endocrinology and Metabolism The Affiliated Hospital of Southwest Medical University Luzhou China; 4 School of Nursing & Public Health Manchester Metropolitan University Manchester United Kingdom; 5 Nursing Faculty Southwest Medical University Luzhou China

**Keywords:** chronic obstructive pulmonary disease, COPD, serious game, rehabilitation, review, mobile phone

## Abstract

**Background:**

The use of serious games for rehabilitation has been an emerging intervention in health care fields, referred to as an entertaining and positive activity. Although related studies have been conducted on patients with chronic obstructive pulmonary disease (COPD), a more comprehensive study that summarizes and evaluates its effects in this area is needed.

**Objective:**

This review aimed to systematically evaluate the effects of serious games in promoting rehabilitation and related outcome measures of serious game–based engagement in patients with COPD.

**Methods:**

This review adhered to the PRISMA (Preferred Reporting Items for Systematic Reviews and Meta-Analyses) statement. Searches were performed in the following databases: PubMed, Scopus, Embase (via Ovid), CINAHL, Science Direct, and China Biology Medicine disc. Only quantitative studies were included in this review, and the methodological quality and bias of the included studies were evaluated using related tools. Several outcomes, including clinical outcomes and serious game–based engagement outcomes, were ultimately collected in this review. The results were summarized and evaluated using descriptive methods due to significant heterogeneity.

**Results:**

In total, 11 studies were included. Serious games played a potentially positive effect on pulmonary function and exercise capacity. However, no consistent findings were reported on dyspnea and psychological status. Additionally, serious game engagement showed favorable findings on adherence, enjoyment, and acceptability. Furthermore, no serious adverse effects were identified in all included studies.

**Conclusions:**

This review preliminarily indicated the potential benefits of serious games in promoting rehabilitation for patients with COPD, despite the limited quality of the included studies. More studies with high methodological quality are needed to further explore the effects of serious games in this field.

## Introduction

### Background

Chronic obstructive pulmonary disease (COPD) is a serious health condition that affects millions of people worldwide. It is characterized by progressive and irreversible obstruction of the airways, which leads to difficulties in breathing, reduced lung function, and increased risk of exacerbations [1]. COPD is currently the most common chronic respiratory disease worldwide [2] accounting for approximately 7% of deaths worldwide [3]. Pulmonary rehabilitation (PR) is an important tailored intervention involving several components that include but are not limited to exercise training, education, and breathing exercise in COPD management, as it can help patients improve their physical function, quality of life, and overall health outcomes [4]. Currently, PR has been widely recommended as an effective treatment but relies on continuous adherence for patients with COPD. However, despite the anticipated benefits of PR, the attendance, adherence, and completion rates of PR in patients with COPD are as low as 70%, 50%, and 40%, respectively [5-7], particularly for those who live in remote or underserved areas [8]. There are several potential barriers stopping patients from performing PR, such as low motivation, a lack of social support, transportation difficulties, and interest [7,9,10]. Hence, it appears essential and crucial to overcome these barriers and provide support for patients with COPD to engage in PR. Serious games, also known as therapeutic games, have emerged as a promising alternative therapy. Serious games encompass a broad concept that entails using distinct game elements to educate or induce changes in experiences or behaviors consisting of diverse forms such as video games and exergames [11]. These games, which may either be designed specifically for health purposes or be designed commercially for leisure exercise, use digital and engaging gameplay to deliver therapeutic interventions and promote healthy behaviors. Serious games have been used to address a wide range of health conditions, including mental health, chronic pain, and stroke rehabilitation [12,13]. In recent years, with the further widespread use of serious games, there has been a growing interest in the use of serious games for assisting patients with COPD in performing PR interventions. One of the potential reasons for the increasing popularity is that serious games may attract patients’ attention away from briefly boring repetition of rehabilitation intervention and enhance their enjoyment [14,15]. Additionally, extra advantages of serious games over traditional PR have also been identified, such as objective data on performance and progress, low cost, enhanced motivation, and adherence [16]. However, the relationship between serious games and effectiveness in patients with COPD still remains poorly understood.

To date, there is only one systematic review that specifically examines the effects of active video games in patients with COPD [17]. However, this review, proving a potentially positive role of video games in terms of adherence, enjoyment, and various clinical outcomes, has limitations. First, it overlooks several relevant studies [18-22] that were published after the review was conducted. Second, the review only focuses on video games and lacks the inclusion of diverse types of serious games. As mentioned before, video games are a narrower category within the broader scope of serious games [23]. Third, the review lacks attention to certain outcome measures, such as pulmonary function and acceptability.

Additionally, 2 other reviews with a broader scope in respiratory conditions (asthma, COPD, emphysema, bronchiectasis, etc) separately reported consistent results in adherence and enjoyment [24] as well as exercise capacity and quality of life [25]. However, another review focused on respiratory diseases claimed no improvement in dyspnea or exercise capability [26].

Based on current evidence, the effects of serious games on patients with COPD remain unclear due to several limitations, including a limited number of enrolled original studies, a restricted scope of serious games, and inadequate and inconsistent reporting of results. A more comprehensive review is needed to further systematically evaluate the available evidence on the effects of health-related serious games for patients with COPD. Therefore, this review aims to include more eligible studies, a wider range of serious game types, and diverse outcomes to offer a comprehensive and unbiased overview of the current state of the evidence and identify areas for future research by using systematic methods.

### Objectives

The overall aim of this review was to evaluate the effects of serious games for two categories of outcomes: (1) clinical outcomes, including pulmonary function, exercise capacity, daily steps, dyspnea, patient-reported outcome measures, and psychological condition, as well as adverse effects, and (2) related outcome measures of serious game–based engagement, including adherence, enjoyment, and acceptability.

## Methods

### Data Sources and Search Strategies

PICO, which stands for Participant, Intervention, Control, Outcome, was the most commonly used framework to guide review questions and facilitate searches [27]. In this review, the modified framework of “PICOs,” which stands for patient problem, intervention, comparison, study of design, was used to explicitly state and derive the search terms that included subject headings and keywords in each part. However, it should be noted that due to differences in the expression forms of subject headings in diverse databases, the subject headings were adapted to fit each database accordingly. Additionally, the search strategy only used “P,” “I,” and “S” to broaden the search scope. The search strategies were separately searched in 6 electronic databases, which were PubMed, Scopus, Embase (via Ovid), CINAHL, Science Direct database, and China Biology Medicine disc, from inception to December 20, 2022. The search was initially undertaken in PubMed and then tailored and applied to the other 6 databases. Furthermore, the reference lists of eligible studies were also screened to identify studies. Multimedia Appendix 1 summarizes the search strategies that were used in all included databases.

### Inclusion and Exclusion Criteria

The inclusion criteria were as follows: (1) quantitative studies, including randomized controlled trials (RCTs) and pre-post studies; (2) studies, in which participants had been clinically diagnosed with COPD without limitations of age, gender, and ethnicity; (3) intervention involving serious games for therapeutic purposes using any electronic platform (tablets, computers, consoles, smartphones, televisions, or any other digital devices); (4) no control groups or the control group used usual care or nonserious games; (5) results included health-related outcomes such as exercise capacity, pulmonary function, dyspnea, or adverse effects, as well as serious game–based engagement outcomes such as adherence, enjoyment, or acceptability; and (6) studies must be peer-reviewed and be in English or Chinese.

The exclusion criteria were (1) duplicate records; (2) studies focused on measurement, diagnostic methods, serious game theory, or game development; (3) nondigital games and serious games used for other purposes, such as screening, or nonhealth educational purposes; or (4) conference abstracts or studies for which full texts could not be derived.

### Study Selection and Data Extraction

Two authors (HH and QC) independently screened the titles and abstracts of relevant studies using EndNote software (version X9; Clarivate Analytics). The full texts of eligible studies were then independently identified by the same authors according to predefined selection criteria. Any conflicts that arose between the 2 authors were resolved through discussion. If a consensus could not be reached, a third author (M Huang) was consulted to make a final decision. A data collection form was created to extract various characteristics of the studies, including the authors, study design, setting, participant demographics, intervention, comparators, frequency of the course, outcome measures, and instruments used. Additionally, a serious game intervention protocol form was developed to summarize the details of the interventions, including the serious game modalities, source of the serious games, procedure of the serious game modality, duration, length, and frequency of the intervention. The data extraction procedure was as follows: 2 authors (HH and QC) independently extracted data using predefined forms. Any discrepancies were addressed through discussion, and a third author (M Huang) was consulted to reach a final decision. Two electronic documents of the extracted data were created and managed by HH and QC.

### Quality Assessment of Included Studies

The Cochrane Library’s RCT assessment tool [28] was used to evaluate the methodological quality and risk of bias in RCTs. This tool consists of 7 domains: sequence generation, allocation concealment, blinding of participants and personnel, blinding of outcome reporting, incomplete outcome data, selective outcome reporting, and other biases. The JBI quasi-RCT assessment tool [29] was used to assess pre-post studies. The assessment of methodological quality and risk of bias was carried out by 2 authors (HH and M Huang), and a third author (QC) was consulted to make a final decision if any discrepancy occurred.

### Data Synthesis

A meta-analysis was initially expected to calculate pooled effects. However, the significant clinical heterogeneity identified in the types of studies, comparisons, intervention protocols, and varied outcomes deemed it an inappropriate method for undertaking quantitative synthesis. Therefore, descriptive analysis was used to summarize the effects of serious games on rehabilitation in patients with COPD. The process of conducting descriptive analysis to present the research results involved tabulating various characteristics, such as participants, intervention details, comparison groups, outcomes, sample size, and study designs, from the original studies. This information was independently extracted in a predefined Excel (Microsoft Corp) form by HH and M Huang. Subsequently, the collected data were thoroughly reviewed and verified by the 2 authors to ensure accuracy and reliability. In case of any disagreements, consensus was reached through discussion. Finally, the data were compared against the planned studies for each specific outcome, facilitating a comprehensive evaluation of the findings. Specifically, the theme of identifying the relationships between serious game interventions and patient outcomes (eg, pulmonary function, exercise capacity, daily steps, dyspnea, psychological status, adverse effects, and measures of game-based engagement) was summarized.

## Results

### Search Results

**Figure 1:**
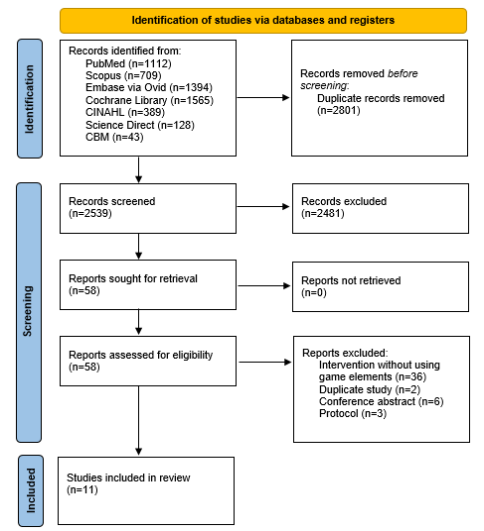
Flowchart of studies screening. CBM: China Biology Medicine.

The database search yielded 5340 publications in total. A total of 2801 duplicate items were excluded using EndNote (version X9), and another 2481 items were further removed after reading through titles and abstracts. The full texts of the remaining studies were retrieved for a more detailed assessment, of which 47 were removed for the following reasons: (1) intervention without using game elements (n=36), (2) duplicate study (n=2), (3) conference abstract (n=6), and (4) study protocol (n=3). As a result, a total of 11 studies were finally included in this review. Figure 1 illustrates a flowchart of the study screening process.

### General Characteristics of Included Studies

The key characteristics of the enrolled studies are shown in Multimedia Appendix 2. This review eventually consisted of 11 studies published between 2011 and 2021 with 8 RCT studies [18-22,30-32] and 3 pre-post studies [33-35]. Of the 11 studies, 3 were published in Chinese [21,22,32], and the remaining were reported in English [18-20,30,31,33-35]. The countries undertaking the interventions varied greatly, including mainland China in 3 studies [21,22,32], Canada in 2 studies [30,35], the United States in 2 studies [33,34], Australia in 1 study [18], Italy in 1 study [31], Poland in 1 study [19], and Indonesia in 1 study [20]. The studies’ sample sizes ranged from 10 to 130 with most studies having fewer than 50 participants, and only 2 studies having sample sizes greater than 100 [19,21]. The mean age of participants was beyond 60 years in all included studies. Regarding the intervention protocols, 10 experimental studies [19-22,30-35] separately used commercial software containing game elements as intervention tools, and the most commonly used software was the Wii Fit system (Nintendo). Only 1 study [18] developed serious game software. The duration of the interventions ranged from 2 to 18 weeks in all experimental studies reporting intervention duration [18-21,31-34]. Meanwhile, the frequencies of using the interventions varied significantly across the studies.

### Methodological Quality and Risk of Bias of the Included Studies

The results of the methodological quality of all studies except the descriptive one are presented in Tables 1 and 2. For RCT studies, randomization allocation was mentioned in all 8 studies, and 6 studies [19-22,30,32] reported the details on random sequence generation using random number tables or computer-generated number sequences or web-based number sequences. Allocation concealment was mentioned in only 1 study via sealed opaque envelopes [19]. Four studies [18-20,31] described blinding of participants and personnel as not applicable, and the remaining studies did not describe that blinding. Only 1 study stated blinding of outcome assessment [19]. All 8 studies stated participants’ dropout rates, and all participants who completed the intervention were incorporated into the data analysis. In addition, all RCT studies were considered low risk of selective outcome reporting. For “other bias,” 3 studies reported a sample size calculation. All studies conducted baseline assessment reporting without significant differences. Inclusion criteria, exclusion criteria, end points, and method of data analysis were clearly reported in all 8 studies. Only 2 studies described adverse events during the period of intervention [21,31]. For pre-post studies [33-35], 3 studies met all criteria except for a comparison group.

**Table 1:** Methodological assessment of the randomized controlled trial studies.

Criteria			Simmich et al [18]		Sutanto et al [20]		Hu et al [32]		LeGear et al [30]		Rutkowski et al [19]		Mazzoleni et al [31]		Zhou et al [21]		Jin et al [22]
Random sequence generation			?^a^		✓^b^		✓		✓		✓		?		✓		✓
Allocation concealment			？		?		?		?		✓		?		?		?
Blinding of participants and personnel			×^c^		×		?		?		×		×		?		?
Blinding of outcome assessment			×		×		?		?		✓		×		?		?
Incomplete outcome data			✓		✓		✓		✓		✓		✓		✓		✓
Selective outcome reporting			✓		✓		✓		✓		✓		✓		✓		✓
**Other bias**			×		✓		×		×		?		✓		×		×
	Sample size calculation	×		✓		×		×		✓		✓		×		×	
	Baseline assessment	✓		✓		✓		✓		✓		✓		✓		✓	
	Inclusion criteria	✓		✓		✓		✓		✓		✓		✓		✓	
	Exclusion criteria	✓		✓		✓		✓		✓		✓		✓		✓	
	Evaluation of therapeutic effect	✓		✓		✓		✓		✓		✓		✓		✓	
	Report of adverse events	?		✓		?		?		?		✓		?		?	
	Method of data analysis	✓		✓		✓		✓		✓		✓		✓		✓	

^a^Unclear.

^b^Low risk.

^c^High risk.

**Table 2:** Methodological assessment of the pre-post studies.

Criteria	Albores et al [33]	Wardini et al [34]	Parent et al [35]
Is it clear in the study what is the “cause” and what is the “effect” (ie, there is no confusion about which variable comes first)?	✓^a^	✓	✓
Were the participants included in any comparisons similar?	N/A^b^	N/A	N/A
Were the participants included in any comparisons receiving similar treatment or care other than the exposure or intervention of interest?	N/A	N/A	N/A
Was there a control group?	×^c^	×	×
Were there multiple measurements of the outcome both pre and post the intervention or exposure?	✓	✓	✓
Was follow-up complete and if not, were differences between groups in terms of their follow-up adequately described and analyzed?	✓	✓	✓
Were the outcomes of participants included in any comparisons measured in the same way?	✓	✓	✓
Were outcomes measured in a reliable way?	✓	✓	✓
Was appropriate statistical analysis used?	✓	✓	✓

^a^Yes.

^b^N/A: not applicable.

^c^No.

### Current Serious Game Intervention Protocols of PR

The serious game intervention protocols used in the included studies are summarized in Table 3, including serious game modalities, source of serious game, the procedure of the serious game modality, duration, length, and frequency of the intervention. Of them, only 1 study [18] developed a serious game modality for the purposes of the study.

**Table 3:** Details of SG^a^ intervention protocol.

Study	SG modalities	Source of SG	Procedure of the SG modality	Duration	Length and frequency
Simmich et al [18]	HW^b^: Fitbit SW^c^: grow stronger AVG app	Purpose-built	Fitbit paired to SW on smartphones and tracked steps, physical activity, and heart rate Game modes on SW (1) Complete and report upper and lower body physical activity, (2) grow a garden, and (3) a cooperative multiplayer game visiting multiple well-known Australian destinations	3 weeks	Each day
Sutanto et al [20]	HW: a balance board accessory SW: game activities of Wii Fit system	Off-the-shelf	Yoga: deep breathing and half moon Strength training Aerobic exercise	6 weeks	30 min per session, 3 times per week
Hu et al [32]	HW: not reported SW: game activities of BioMaster virtual reality digital rehabilitation training system	Off-the-shelf	Upper body physical activities: housework activities, kitchen cooking, etc Lower body physical activity: cycling	12 weeks	5-15 min per day
LeGear et al [30]	HW: a balance board accessory SW: game activities of Wii Fit system	Off-the-shelf	Marching, dancing, and 2 air punching exercises in sequence	N/A^d^	15 min per session
Rutkowski et al [19]	HW: the console, a Kinect motion sensor, and a projector with speakers SW: game activities of Kinect somatosensory digital games	Off-the-shelf	Rafting, cross-country running, hitting a ball in the direction of a player on the screen, and a mountain wagon ride	2 weeks	20 min per day, 5 days per week
Mazzoleni et al [31]	HW: haptic sensor–based controllers and a balance board SW: game activities of Wii Fit Plus	Off-the-shelf	Two 5-min sessions of “yoga” activity consisting of deep breathing Jogging Plus consisting of feedback aided 10-min running Twisting and squat: 10-min trunk twisting and arm and leg squatting	1 week	60 min per day
Zhou et al [21]	HW: console, Kinect motion sensor, and projector with speakers SW: game activities of Kinect somatosensory digital games	Off-the-shelf	Trail running, rafting, hitting, and mountain riding in sequence	18 weeks	30 min per day, 5 days per week
Jin et al [22]	HW: remote controls SW: game activities of somatosensory digital games developed by Chinese company	Off-the-shelf	Kitchen knife, ping pong exercise, and swimming in sequence	Admission within 48 h to discharge from hospital	20 min per day
Albores et al [33]	HW: haptic sensor–based controllers and a balance board SW: game activities of Wii Fit unit	Off-the-shelf	Basic run: warm-up exercise Bird’s eye Bull’s eye: mostly upper arm exercise Free step: mostly lower extremity exercise Obstacle course: upper and lower extremity exercise Basic run: cool-down exercise	12 weeks	5 or more days per week
Wardini et al [34]	HW: a balance board accessory SW: game activities of Wii Fit system	Off-the-shelf	Basic run, basic step, obstacle course, and island cycling for lower body training Boxing and canoeing for upper body training	3 to 4 weeks	3 times per week
Parent et al [35]	HW: a console and a controller SW: game activities of Kinect	Off-the-shelf	A running game (Stunt Run), a boxing game (Arctic Punch), a core twist game (To the Core), and a squat game (Squat Me to the Moon) in sequence	N/A	30 min per session

^a^SG: serious game.

^b^HW: hardware.

^c^SW: software.

^d^N/A: not applicable.

### Commercial Game Software Combined With Hardware

In total, 10 studies [19-22,30-35] used commercial serious game systems, of which 5 [20,30,31,33,34] used Wii game systems (Nintendo), 3 studies [19,22,35] used Kinect game systems (PrimeSense), 1 study [22] used a Chinese brand game system, and the remaining [32] used the BioMaster virtual reality digital rehabilitation training system. Serious game modalities in each study comprised 2 key components, hardware and software except one study [32] that did not report the details of hardware. Specifically, software provided game contexts and images, whereas hardware was responsible for capturing and conducting movements in the games. The procedure of the serious game modality greatly varied from different studies, but all included multiple sessions of games in regard to training the upper body, lower body, or breathing exercises. In total, 7 studies reported that the duration of the interventions varied from 2 to 18 weeks [19-21,31-34]. However, 2 studies [30,35] did not report the duration of the intervention, and 1 study [22] only reported the duration from admission within 48 hours to discharge, which made it impossible to confirm the duration. Regarding the length and frequencies of undertaking serious games across the studies, a consensus was not reached in the trials that reported this parameter, ranging between 5 and 60 minutes per day in length and between 3 days per week and each day in frequency.

### Purpose-Built Game Software Combined With Hardware

Only 1 study [18] specifically developed its serious game modality combined with a Fitbit and games that was produced by a combination of researchers, patients with COPD, and clinicians with experience in PR. Fitbit was used to track steps, physical activity, and heart rate in participants with COPD, whereas a game with 2 game modes was developed to guide physical training. The duration of intervention was reported to be 3 weeks, and the frequency was just briefly stated as every day.

### Relationships Between Serious Game Interventions and Patient Outcomes

The results of serious game interventions were presented using descriptive analysis for this review.

### Pulmonary Function

In total, 2 of the 12 studies separately examined the outcomes of the index of pulmonary function using FEV (forced expiratory volume) 1%predicted and FEV1/FVC (forced vital capacity) at different time points. FEV1%predicted, indicating the severity of diseases, refers to FEV in 1 second predicted, and FEV1/FVC is the ratio of the amount of air that a person can forcefully exhale in 1 second (FEV1) to the total amount of air that can be exhaled (FVC) over the course of a full exhalation, revealing that the airways are obstructed or narrowed for respiratory diseases [36]. One study [32] comparing the BioMaster virtual system with the conventional PR program showed no significant difference in FEV1%predicted and FEV1/FVC at 4 weeks but presented significant improvements at 8 weeks and 12 weeks after the intervention, respectively (P<.05). Similarly, another study [21] reported consistent results in which the somatosensory game digital training group had a significant improvement compared with the traditional exercise training group after 18 weeks of the intervention.

### Exercise Capacity

In total, 7 studies [19-22,31-33] examined physical exercise capacity with varied measurements, of which 5 [19-21,31,32] used a 6-minute walk test, 1 [22] used a brief balance evaluation system test, 1 [33] adopted the endurance shuttle walk test and arm-lift and sit-to-stand repetitions, and 1 [19] applied senior fitness test. Additionally, the protocols between groups highly varied, which were BioMaster virtual system versus the conventional PR program [32], Wii versus exercise training on a cycle ergometer [20], Wii versus the conventional PR program [31], somatosensory game digital training versus traditional exercise training [21], somatosensory game digital training versus usual care [22], endurance exercise training versus virtual reality training [19], and Wii only [33]. Four studies separately showed significant improvements in the 6-minute walk test at 1 [31], 2 [19], 12 [32], and 18 weeks [22], whereas 1 study [20] showed a reverse result at 6 weeks of the intervention between groups. In addition, the results in 2 studies showed a significant improvement between groups in the senior fitness test at 2 weeks [19], the endurance shuttle walk test and arm-lift and sit-to-stand repetitions at 12 weeks [33]. Furthermore, 1 study [22] found a significant improvement in the brief balance evaluation system test of somatosensory digital games from admission within 48 hours to discharge from the hospital of the intervention compared with the usual care group.

### Daily Steps

Only 1 study [18] collected continuous data on daily steps using Fitbit devices. The protocol involved purpose-built serious games combined with Fitbit in the experimental group and Fitbit alone in the control group [18]. The result stated that participants in both groups had high adherence to wearing Fitbit, recording steps on >80% of days, and showed opposite trends between the 2 groups in daily steps across 3 weeks, which indicated a significant improvement in the experimental group and a decrease in the control group [18].

### Dyspnea

In total, 6 studies [20,21,30,31,34,35] evaluated dyspnea using 2 different tools, of which 4 studies [21,30,34,35] used the Borg dyspnea scale, and the remaining 2 studies [20,31] used the Medical Research Council score. Currently, there are conflicting results. Specifically, 2 RCTs [21,30] in the Borg dyspnea scale and 2 RCTs [20,31] in the Medical Research Council reported no significant improvements between groups, and 1 pre-post study [35] also obtained a similar result in the Borg dyspnea scale. However, another pre-post study [34] presented an opposite result that showed a significant improvement after intervention in the Borg dyspnea scale.

### Patient-Reported Outcome Measures and Psychological Evaluation

In total, 3 studies [20,31,33] evaluated psychological status, including quality of life with the Saint George’s Respiratory Questionnaire in 2 studies [20,31] and the Chronic Respiratory Questionnaire in 1 study [33], and depression with the Beck Depression Inventory in 1 study [31]. Two studies [20,31] showed no significant improvements in Saint George’s Respiratory Questionnaire between groups, whereas 1 study had the opposite result in which there was a significant increase in Chronic Respiratory Questionnaire after the intervention. In terms of depression, no statistically significant improvement in Beck Depression Inventory was identified between groups [31].

### Adverse Effects

Most studies did not report adverse effects, but only 2 studies [33,34] revealed chest pain and decreased oxygen saturation in the process of intervention. No serious adverse effects were identified in any of the studies.

### Related Outcome Measures of Serious Game–Based Engagement

#### Adherence and Use of Serious Games

Two studies [21,34] reported adherence, which was highly varied. The adherence rate was 64% in a pre-post study [34] to test Wii serious games with an intervention duration of 3-4 weeks, whereas it accounted for 83.61% in an RCT study [21] to evaluate somatosensory game digital training after 18 weeks. One study reported game use frequency as participants who were involved in the study automatically recorded activities on 58.6% (82/141) of the days during the 3-week intervention [18]. Additionally, another study obtained adherence data from self-reported patient diaries, reporting that patients used serious games for a total of 36 hours and 5.7 days per week with a mean number of hours per week ranging from 2.6 to 3.4 [33].

#### Enjoyment

Four studies reported outcome measures of enjoyment of serious games with different instruments [18,30,34,35]. One study [30] used a 5-question Likert scale to evaluate enjoyment and found that 90% of participants enjoyed using the Wii exercise program. Similarly, another study [35] assessed the enjoyment of Kinect game activities using a 5-question Likert scale and reported consistent results that participants were more likely to enjoy the games. Moreover, a 10-cm visual analog scale was used to measure overall enjoyment based on the Wii Fit system [34]. The results demonstrated that participants had a mean score of 8 out of 10. Participants also reported a mean score of 8 out of 10 when asked about their willingness to recommend serious games to another patient with COPD. Additionally, a subscale on the Intrinsic Motivation Inventory was used to test enjoyment with a mean score of 5.4 out of 7 [18]. In summary, participants in these studies found serious games enjoyable.

#### Acceptability

Only 1 study [31] reported using a Likert scale to test the acceptability of serious games. This study found a higher score of 42.4 out of 49 in the group that participated in digital video game–aided exercises, which was similar to the score of the standard PR group. However, no significant difference was identified between the 2 groups.

## Discussion

### Principal Findings

To the best of our knowledge, this review is not the first to systematically investigate the effects in this field. However, it provides a broader insight, contributing to the understanding of the effects of serious games by rigorously examining a greater number of original studies, various types of serious games, and outcomes. This review indicated a promising role of serious games in the rehabilitation of patients with COPD. Nevertheless, the findings should be interpreted carefully considering that relevant evidence on serious games in this area was deemed not fully conclusive due to the wide variation in the design of studies, serious game modality, characteristics and duration, study comparisons, outcomes, and high risks of bias identified in some included studies.

The findings showed that serious games could be beneficial to pulmonary function, indicating that serious games may be a potentially effective alternative or complement to traditional rehabilitation in improving pulmonary function for individuals with COPD. It is commonly believed that serious games can be tailored to the individual needs of the patient, allowing for more personalized and effective treatment [37]. Additionally, the use of serious games can provide a more engaging and digital experience for patients, which may lead to increased adherence and motivation to exercise [38]. Therefore, increased physical exercise in intervention groups may contribute to better pulmonary function compared to control groups. However, it is important to note that more research is needed to examine the long-term effects of serious games on pulmonary function.

The findings in this review indicated a potentially beneficial role of serious games on exercise capacity. Most studies reported consistent results in favor of significant improvement when using serious games compared to the control group, which was in line with the findings of a previous review [17]. Furthermore, continuous monitoring data on daily steps also supported the effectiveness of serious games [18]. It is likely that participation in serious games provides a more engaging and digital form of exercise, which motivates patients and facilitates engagement [38]. This has been identified as a key factor in increasing adherence to interventions [39-42]. Additionally, compared to traditional forms of exercise as walking on a treadmill or cycling on a stationary bike, serious games are less boring and more appealing to patients, providing explicit instructions for patients to perform physical exercises as well [43].

It appeared that current findings have not found evidence that serious games have a positive impact on dyspnea in patients with COPD. Most studies [20,21,30,31,34,35] included in the review focused on physical exercises combined with serious games rather than using breathing techniques or exercises. In addition, the findings may be limited by varied measurement tools and the quality of studies. Therefore, it remains unclear whether serious games alone have any impact on dyspnea in patients with COPD. Additional research that specifically examines the use of breathing techniques or exercises in serious games may provide more insights into potential benefits for patients with COPD in the future.

Findings regarding the impact of serious games on quality of life and depression in patients with COPD were inconclusive, which indicates there was no consistent improvement observed in these areas and serious games may not have a significant impact on psychological status when compared to other interventions [20,31,33]. It should be highlighted that improvements in human well-being and depression are probably influenced by multiple factors [44,45], and the studies included in this review primarily focused on physical levels rather than other components, such as health education, and self-management. This may result in the lack of observed improvements. Additionally, it is unclear whether significant improvements would be found with long-term intervention.

Safety outcomes associated with serious games were not reported in the majority of studies included in this review. Only 2 studies [33,34] described adverse effects, including chest pain and decreased oxygen saturation. This suggests that potential adverse reactions have not yet been fully identified, highlighting the need for future research to focus on evaluating the safety outcomes of serious games and identifying any potential adverse effects that may occur.

Current findings on serious games’ impact on adherence, enjoyment, and acceptability highlighted positive outcomes. It is recognized that PR entails numerous benefits; however, adhering to the program is currently one of the most significant challenges [4,44,45]. Insights into patients’ continuous attendance are essentially significant, and both patients’ acceptability and enjoyment are valuable indicators of this [46,47]. The current studies [18,30,34,35] have reported a high level of enjoyment, whereas only one study [31] has mentioned acceptability, which was found to be higher in the serious games group. These findings may be attributed to the concept that serious games provide a fun virtual environment for the patients, combining physical exercises with games to make the patients have fun, thus improving patient enjoyment and acceptability [17]. Then, the high level of acceptability and enjoyment contributes to good patient adherence, as reported in this review. Additionally, the findings in this review also show that the somatosensory game digital system, which uses body movements and gestures as input, had a higher adherence rate than the Wii serious games system, which relies solely on hand-held controllers for interaction [21]. This suggests that the use of a more immersive and physically engaging system may lead to increased participation and engagement from users, resulting in a higher adherence rate. This highlights the importance of considering the type of digital system used in serious game interventions, as it may have a significant impact on the effectiveness of the intervention [11]. Additionally, the adherence rate may vary depending on the population and specific intervention being used [48], and further research is needed to fully understand the impact of digital systems on adherence rates in serious game interventions.

Significant variation involving intervention modality, the procedure of the intervention modality, duration, length, and frequency was observed in all studies. Commercial serious game systems were the most frequently used modalities compared to purpose-built game systems among all studies. In addition, Wii game systems involving economic cost were the most popular in all included studies. Additionally, the intervention modalities varied greatly among studies. Furthermore, significant variation in duration, length, and frequency was also identified. The considerable variation in serious game protocols among the included studies indicated that an optimal evidence-based serious game protocol in this area is lacking, and further study is therefore warranted to recognize the best available evidence to develop a research-based serious game protocol to promote rehabilitation.

### Comparison to Prior Work

In comparison with the previous review [17], this study effectively addresses the limitation of omitting relevant studies published after its completion [18-22] by incorporating them into the analysis. Moreover, unlike the previous review, which exclusively concentrated on video games, this study embraces a more comprehensive range of serious games, acknowledging that video games represent only a subset of the broader category [23]. Furthermore, this review systematically considers supplementary outcome measures, such as pulmonary function and acceptability, which were inadequately addressed in the earlier review.

### Strengths and Limitations

#### Strengths

This review followed the PRISMA (Preferred Reporting Items for Systematic Reviews and Meta-Analyses) reporting guideline (Multimedia Appendix 3) [49]; thus, it can be viewed as a transparent and reproducible review. The findings in this review are more likely to be comprehensive and provide implications for further understanding the effects of serious games in this area. There is a low risk of publication bias in this review, as the search was conducted to identify as many eligible studies as possible by searching relevant databases using a broad search strategy. Additionally, the risk of selection bias in this review is minimal, as the research process, including study selection, data extraction, and assessment of study quality, was carried out independently by 2 reviewers.

#### Limitations

Although this review identified that serious games are encouraging interventions for promoting rehabilitation in patients with COPD, research evidence of the effects remains inconclusive due to the following limitations in this review and the included studies. First, meta-analysis was not conducted in this review given the significant clinical heterogeneity of the included studies in terms of study design, serious game characteristics, comparisons, and duration, which was one of the main reasons for the inconclusive research evidence found in this review. Second, it is likely that language bias may exist due to the enrollment of only English and Chinese studies in this review. Third, methodological limitations were also found in the included studies. For example, most studies in RCT designs with a small sample size lacked sample size calculations, random allocation of participants, and the use of blinding methods. For pre-post studies, the present studies were limited by the sample size and the absence of an intervention and control group with traditional intervention. Fourth, the effects of serious games involving other vital rehabilitation components in patients with COPD, such as breathing exercises and disease-related health education, are lacking.

However, these limitations mentioned earlier, which could not be addressed in this review, provide significant implications for further studies to enhance the evidence on serious games in this field.

### Implications for Future Research and Practices

An evidence-based serious game protocol should be developed to improve the health of patients with COPD, including an appropriate serious game modality tailored to personalized demands, optimal duration, and frequency, based on the research evidence. Additionally, rigorously designed, large-scale RCTs should be developed to determine the effects of serious games on rehabilitation in patients with COPD, such as random distribution, allocation concealment, and the use of blinding. Moreover, future research should also focus on exploring the effects of serious games on other components of COPD PR, such as breathing exercises or health education in patients with COPD, as knowledge in this area is still insufficient. Furthermore, a more robust systematic review, which includes higher quality studies, addresses language bias, and uses a meta-analysis method, is warranted in the future.

### Conclusions

This review comprehensively summarized and indicated the potentially beneficial role of serious games in enhancing outcomes in patients with COPD. However, these findings should be interpreted with caution due to the unsatisfactory quality of the included studies. It is recommended that more research with rigorous methodological quality is necessary to further determine the role of serious games in promoting rehabilitation.

## Appendix

Multimedia Appendix 1 Search strategies in databases.

Multimedia Appendix 2 General characteristics of included studies.

Multimedia Appendix 3 PRISMA (Preferred Reporting Items for Systematic Reviews and Meta-Analyses) checklist.

## Abbreviations

COPD: chronic obstructive pulmonary disease
FEV: forced expiratory volume
FVC: forced vital capacity
PR: pulmonary rehabilitation
PRISMA: Preferred Reporting Items for Systematic Reviews and Meta-Analyses
RCT: randomized controlled trial
