# Supplementary material for: Effects of Serious Games for Patients With Chronic Obstructive Pulmonary Disease: Systematic Literature Review
Source: JMIR Serious Games. 2023 Sep 25;11:e46358. doi: 10.2196/46358 (PMC10562969; doi:10.2196/46358)
Supplement: Multimedia Appendix 1 [file games_v11i1e46358_app1.docx]

[**Multimedia Appendix 1.**](https://games.jmir.org/api/download?filename=3608428074b980f8e3d8f87984f35e87.docx&alt_name=46358-800078-1-SP.docx) **Search strategies in databases**

**Database(s)：PubMed**

| **ID** | **Searches** | **Results** |
| --- | --- | --- |
| #1 | ((("Pulmonary Disease, Chronic Obstructive"[Mesh]) OR ("Lung Diseases, Obstructive"[Mesh])) OR ("Pulmonary Emphysema"[Mesh])) OR ("Bronchitis, Chronic"[Mesh]) | 228,605 |
| #2 | (((((((((chronic airways obstruction*[Title/Abstract]) OR (Chronic Obstructive Pulmonary Disease[Title/Abstract])) OR (Airflow Obstructio*,Chronic[Title/Abstract])) OR (Obstructive Pulmonary Disease*[Title/Abstract])) OR (Pulmonary Disease*, Obstructive[Title/Abstract])) OR (COPD[Title/Abstract])) OR (COAD[Title/Abstract])) OR (Chronic Obstructive Lung Disease[Title/Abstract])) OR (Chronic Obstructive Airway Disease[Title/Abstract])) OR (chronic airways limitation[Title/Abstract]) | 90,434 |
| #3 | #1 OR #2 | 262,574 |
| #4 | ((((((((("Wireless Technology"[Mesh]) OR "Cell Phone"[Mesh])) OR "Mobile Health Units"[Mesh]) OR "Remote Consultation"[Mesh]) OR "Telerehabilitation"[Mesh]) OR "Video Games"[Mesh]) OR "Internet"[Mesh]) OR "Telemedicine"[Mesh]) OR "Telecommunications"[Mesh] | 208,883 |
| #5 | (((((((((((((((((((((((Telehealth* [Title/Abstract]) OR (telecare* [Title/Abstract])) OR (telehomecare* [Title/Abstract])) OR (telehealthcare* [Title/Abstract])) OR (telemonitor*[Title/Abstract])) OR (telemanagement* [Title/Abstract])) OR (telematic*[Title/Abstract])) OR (telesupport* [Title/Abstract])) OR (web based[Title/Abstract])) OR (Phone* [Title/Abstract])) OR (wireless* [Title/Abstract])) OR (mHealth[Title/Abstract])) OR (ehealth[Title/Abstract])) OR (mobile Health[Title/Abstract])) OR (mobile healthcare* [Title/Abstract])) OR (m healthcare* [Title/Abstract])) OR (home care* [Title/Abstract])) OR (home healthcare* [Title/Abstract])) OR (interactive* [Title/Abstract])) OR (virtual* [Title/Abstract])) OR (remote[Title/Abstract])) OR (video* [Title/Abstract])) OR (APP* [Title/Abstract])) OR (telenursing[Title/Abstract]) | 474,615 |
| #6 | (((((((((((((((((((serious game*[Title/Abstract]) OR (health game[Title/Abstract])) OR (game[Title/Abstract])) OR (video game*[Title/Abstract])) OR (playthings[Title/Abstract])) OR (smartphone game*[Title/Abstract])) OR (mobile phone game[Title/Abstract])) OR (computer game*[Title/Abstract])) OR (digital game[Title/Abstract])) OR (gamification[Title/Abstract])) OR (virtual game*[Title/Abstract])) OR (online game*[Title/Abstract])) OR (applied game*[Title/Abstract])) OR (simulation game*[Title/Abstract])) OR (Virtual reality game*[Title/Abstract])) OR (VR game*[Title/Abstract])) OR (Augmented reality game*[Title/Abstract])) OR (Mobile game*[Title/Abstract])) OR (Television game*[Title/Abstract])) OR (TV game*[Title/Abstract]) | 34,258 |
| #7 | #4 OR #5 OR #6 | 638,153 |
| #8 | (((((((((((randomized controlled trial[Title/Abstract]) OR (RCT[Title/Abstract])) OR (randomized[Title/Abstract])) OR (evidence-based[Title/Abstract])) OR (follow up[Title/Abstract])) OR (intervention study[Title/Abstract])) OR (single-blind method[Title/Abstract])) OR (double-blind method[Title/Abstract])) OR (random allocation[Title/Abstract])) OR (intervention study[Title/Abstract])) OR (clinical trial[Title/Abstract]) | 1,908,291 |
| #9 | #3 AND #7 AND #8 | 1,112 |

**Database(s):**Embase via Ovid

| **ID** | **Searches** | **Results** |
| --- | --- | --- |
| #1 | Pulmonary Disease, Chronic Obstructive.mp. or exp chronic obstructive lung disease/ | 154330 |
| #2 | Lung Disease, Obstructive.mp. | 51 |
| #3 | Pulmonary Emphysema.mp. or lung emphysema/ | 14275 |
| #4 | Bronchitis, Chronic.mp. or exp chronic bronchitis/ | 10677 |
| #5 | (obstructive lung disease* or chronic airways obstruction* or Chronic Obstructive Pulmonary Disease or Airflow Obstructio*, Chronic or Obstructive Pulmonary Disease* or Pulmonary Disease*, Obstructive or COPD or COAD or Chronic Obstructive Lung Disease or Chronic Obstructive Airway Disease or chronic airways limitation).ab,ti. | 133475 |
| #6 | 1 or 2 or 3 or 4 or 5 | 197645 |
| #7 | exp telecommunication/ | 98131 |
| #8 | exp telemedicine/ | 59723 |
| #9 | exp Internet/ | 121189 |
| #10 | exp wireless communication/ | 6595 |
| #11 | exp mobile phone/ | 39533 |
| #12 | Mobile Health Units.mp. | 77 |
| #13 | exp teleconsultation/ | 13575 |
| #14 | exp telerehabilitation/ | 1787 |
| #15 | exp video game/ | 5088 |
| #16 | (Telehealth or telecare or telehomecare or telehealthcare or telemonitor* or telemanagement or telematic* or telesupport or web based or Phone or wireless or mHealth or ehealth or mobile Health or mobile healthcare or m healthcare or home care or home healthcare or interactive or virtual or remote or video or APP or telenursing or serious game* or health game or game or video game* or playthings or smartphone game* or mobile phone game or computer game* or digital game or gamification or virtual game* or online game* or applied game* or simulation game* or Virtual reality game* or VR game* or Augmented reality game* or Mobile game* or Television game* or TV game*).ab,ti. | 630625 |
| #17 | 7 or 8 or 9 or 10 or 11 or 12 or 13 or 14 or 15 or 16 | 796740 |
| #18 | (randomized controlled trial or RCT or randomized or evidence-based or follow up or intervention study or single-blind method or double-blind method or random allocation or intervention study or qualitative study or clinical trial).ab,ti. | 2833541 |
| #19 | 6 and 17 and 18 | 1394 |

**Database(s):**Cochrane library

| **ID** | **Searches** | **Results** |
| --- | --- | --- |
| #1 | (obstructive lung disease* OR chronic airways obstruction* OR Chronic Obstructive Pulmonary Disease OR Airflow Obstructio*,Chronic OR Obstructive Pulmonary Disease* OR Pulmonary Disease*, Obstructive OR COPD OR COAD OR Chronic Obstructive Lung Disease OR Chronic Obstructive Airway Disease OR chronic airways limitation):ti,ab,kw | 23538 |
| #2 | MeSH descriptor: [Pulmonary Disease, Chronic Obstructive] this term only | 5945 |
| #3 | MeSH descriptor: [Lung Diseases, Obstructive] this term only | 3132 |
| #4 | #1 OR #2 OR #3 | 23538 |
| #5 | MeSH descriptor: [Telecommunications] this term only | 89 |
| #6 | MeSH descriptor: [Telemedicine] this term only | 2713 |
| #7 | MeSH descriptor: [Internet] this term only | 4165 |
| #8 | MeSH descriptor: [Wireless Technology] this term only | 45 |
| #9 | MeSH descriptor: [Cell Phone] this term only | 790 |
| #10 | MeSH descriptor: [Mobile Health Units] this term only | 68 |
| #11 | MeSH descriptor: [Remote Consultation] this term only | 387 |
| #12 | MeSH descriptor: [Telerehabilitation] this term only | 170 |
| #13 | MeSH descriptor: [Video Games] this term only | 806 |
| #14 | (Telehealth OR telecare OR telehomecare OR telehealthcare OR telemonitor* OR telemanagement OR telematic* OR telesupport OR web based OR Phone OR wireless OR mHealth OR ehealth OR mobile Health OR mobile healthcare OR m healthcare OR home care OR home healthcare OR interactive OR virtual OR remote OR video OR APP OR telenursing OR serious game* OR health game OR game OR video game* OR playthings OR smartphone game* OR mobile phone game OR computer game* OR digital game OR gamification OR virtual game* OR online game* OR applied game* OR simulation game* OR Virtual reality game* OR VR game* OR Augmented reality game* OR Mobile game* OR Television game* OR TV game*):ti,ab,kw | 94493 |
| #15 | #5 OR #6 OR #7 OR #8 OR #9 OR #10 OR #11 OR #12 OR #13 OR #14 | 96771 |
| #16 | (randomized controlled trial OR RCT OR randomized OR evidence-based OR follow up OR intervention study OR single-blind method OR double-blind method OR random allocation OR intervention study OR qualitative study OR clinical trial):ti,ab,kw in Trials | 1260864 |
| #17 | #4 AND #15 AND #16 | 1565 |

**Database(s):**CINAHL

| **ID** | **Searches** | **Results** |
| --- | --- | --- |
| #1 | DE "OBSTRUCTIVE lung diseases" DE "CHRONIC obstructive pulmonary disease" | 1,160,557 |
| #2 | (DE "PULMONARY emphysema") OR (DE "CHRONIC bronchitis") | 3,311 |
| #3 | AB obstructive lung disease* OR AB chronic airways obstruction* OR AB Airflow Obstructio*,Chronic OR AB Obstructive Pulmonary Disease* OR AB Pulmonary Disease*, Obstructive OR AB COPD OR AB COAD OR AB Chronic Obstructive Lung Disease OR AB Chronic Obstructive Airway Disease OR AB chronic airways limitation | 36,444 |
| #4 | #1 OR #2 OR #3 | 68,101 |
| #5 | DE "TELEMEDICINE" | 18,810 |
| #6 | DE "INTERNET" | 78,312 |
| #7 | ((((DE "WIRELESS communications") OR (DE "CELL phones")) OR (DE "MOBILE health")) OR (DE "COMPUTER consultants")) AND (DE "SONY Vita (Video game console)" OR DE "VIDEO game design") | 23,125 |
| #8 | AB Telehealth OR telecare OR telehomecare OR telehealthcare OR telemonitor* OR telemanagement OR telematic* OR telesupport OR web based OR Phone OR wireless OR mHealth OR ehealth OR mobile Health OR mobile healthcare OR m healthcare OR home care OR home healthcare OR interactive OR virtual OR remote OR video OR APP OR telenursing OR serious game* OR health game OR game OR video game* OR playthings OR smartphone game* OR mobile phone game OR computer game* OR digital game OR gamification OR virtual game* OR online game* OR applied game* OR simulation game* OR Virtual reality game* OR VR game* OR Augmented reality game* OR Mobile game* OR Television game* OR TV game* | 1,042,903 |
| #9 | #5 OR #6 OR #7 OR #8 | 1,116,325 |
| #10 | AB randomized controlled trial OR RCT OR randomized OR evidence-based OR follow up OR intervention study OR single-blind method OR double-blind method OR random allocation OR intervention study OR qualitative study OR clinical trial | 1,058,811 |
| #11 | #4 AND #9 AND #10 | 389 |

**Database(s):**CBM

| **ID** | **Searches** | **Results** |
| --- | --- | --- |
| #1 | 慢阻肺 OR 慢性阻塞性肺疾病 OR 肺气肿 OR COPD | 12566 |
| #2 | 严肃游戏 OR 教育游戏OR 游戏模拟 OR 游戏培训 OR游戏 OR视频游戏 OR 任天堂 OR VR OR 虚拟视频 OR 虚拟游戏 OR 游戏化 | 782 |
| #3 | #1 AND #2 | 43 |

| **Databases** | **Searches** | **Results** |
| --- | --- | --- |
| Scopus | ( TITLE-ABS ( "randomized controlled trial" )  OR  TITLE-ABS ( rct )  OR  TITLE-ABS ( randomized )  OR  TITLE-ABS ( evidence-based )  OR  TITLE-ABS ( "follow up" )  OR  TITLE-ABS ( "intervention study" )  OR  TITLE-ABS ( "single-blind method" )  OR  TITLE-ABS ( "double-blind method" )  OR  TITLE-ABS ( "random allocation" )  OR  TITLE-ABS ( "intervention study" )  OR  TITLE-ABS ( "qualitative study" )  OR  TITLE-ABS ( "clinical trial" ) )  AND  ( ( TITLE-ABS ( telecommunications )  OR  TITLE-ABS ( telemedicine )  OR  TITLE-ABS ( internet )  OR  TITLE-ABS ( "Wireless Technology" )  OR  TITLE-ABS ( "Cellular Phone" )  OR  TITLE-ABS ( "Mobile Health Units" )  OR  TITLE-ABS ( "Remote Consultation" )  OR  TITLE-ABS ( telerehabilitation )  OR  TITLE-ABS ( "video game" )  OR  TITLE-ABS ( telehealth )  OR  TITLE-ABS ( telecare )  OR  TITLE-ABS ( telehomecare )  OR  TITLE-ABS ( telehealthcare )  OR  TITLE-ABS ( telemonitor* )  OR  TITLE-ABS ( telemanagement )  OR  TITLE-ABS ( telematic* )  OR  TITLE-ABS ( telesupport )  OR  TITLE-ABS ( "web based" )  OR  TITLE-ABS ( phone )  OR  TITLE-ABS ( wireless )  OR  TITLE-ABS ( mhealth )  OR  TITLE-ABS ( ehealth )  OR  TITLE-ABS ( "mobile Health" )  OR  TITLE-ABS ( "mobile healthcare" )  OR  TITLE-ABS ( "m healthcare" )  OR  TITLE-ABS ( "home care" )  OR  TITLE-ABS ( "home healthcare" )  OR  TITLE-ABS ( interactive )  OR  TITLE-ABS ( virtual )  OR  TITLE-ABS ( remote )  OR  TITLE-ABS ( video )  OR  TITLE-ABS ( app )  OR  TITLE-ABS ( telenursing )  OR  TITLE-ABS ( "serious game*" )  OR  TITLE-ABS ( "health game" )  OR  TITLE-ABS ( game )  OR  TITLE-ABS ( "video game*" )  OR  TITLE-ABS ( playthings )  OR  TITLE-ABS ( "smartphone game*" )  OR  TITLE-ABS ( "mobile phone game" )  OR  TITLE-ABS ( "computer game*" )  OR  TITLE-ABS ( "digital game" )  OR  TITLE-ABS ( gamification )  OR  TITLE-ABS ( "virtual game*" )  OR  TITLE-ABS ( "online game*" )  OR  TITLE-ABS ( "applied game*" )  OR  TITLE-ABS ( "simulation game*" )  OR  TITLE-ABS ( "Virtual reality game*" )  OR  TITLE-ABS ( vr  AND  game* )  OR  TITLE-ABS ( "Augmented reality game*" )  OR  TITLE-ABS ( "Mobile game*" )  OR  TITLE-ABS ( "Television game*" )  OR  TITLE-ABS ( tv  AND  game* ) ) )  AND  ( TITLE-ABS ( "Pulmonary Disease, Chronic Obstructive" )  OR  TITLE-ABS ( "Lung Disease, Obstructive" )  OR  TITLE-ABS ( "Pulmonary Emphysema" )  OR  TITLE-ABS ( "Bronchitis, Chronic" )  OR  TITLE-ABS ( "obstructive lung disease*" )  OR  TITLE-ABS ( "chronic airways obstruction*" )  OR  TITLE-ABS ( "Chronic Obstructive Pulmonary Disease" )  OR  TITLE-ABS ( "Airflow Obstructio*,Chronic" )  OR  TITLE-ABS ( "Obstructive Pulmonary Disease*" )  OR  TITLE-ABS ( "Pulmonary Disease*, Obstructive" )  OR  TITLE-ABS ( copd )  OR  TITLE-ABS ( coad )  OR  TITLE-ABS ( "Chronic Obstructive Lung Disease" )  OR  TITLE-ABS ( "Chronic Obstructive Airway Disease" )  OR  TITLE-ABS ( "chronic airways limitation" ) ) | 709 |
| Science Direct | (obstructive lung disease* OR chronic airways obstruction* OR Chronic Obstructive Pulmonary Disease OR Airflow Obstructio*, Chronic OR Obstructive Pulmonary Disease* OR Pulmonary Disease*, Obstructive OR COPD OR COAD OR Chronic Obstructive Lung Disease OR Chronic Obstructive Airway Disease OR chronic airways limitation) AND (telehealth OR telecare OR telehomecare OR telehealthcare OR telemonitor* OR telemanagement OR telematic* OR telesupport OR web based OR Phone OR wireless OR mHealth OR ehealth OR mobile Health OR mobile healthcare OR m healthcare OR home care OR home healthcare OR interactive OR virtual OR remote OR video OR APP OR telenursing OR serious game* OR health game OR game OR video game* OR playthings OR smartphone game* OR mobile phone game OR computer game* OR digital game OR gamification OR virtual game* OR online game* OR applied game* OR simulation game* OR Virtual reality game* OR VR game* OR Augmented reality game* OR Mobile game* OR Television game* OR TV game*) AND (randomized controlled trial OR RCT OR randomized OR evidence-based OR follow up OR intervention study OR single-blind method OR double-blind method OR random allocation OR intervention study OR qualitative study OR clinical trial) | 128 |
