# Supplementary material for: Effects of Serious Games for Patients With Chronic Obstructive Pulmonary Disease: Systematic Literature Review
Source: JMIR Serious Games. 2023 Sep 25;11:e46358. doi: 10.2196/46358 (PMC10562969; doi:10.2196/46358)
Supplement: Multimedia Appendix 2 [file games_v11i1e46358_app2.docx]

**[Multimedia Appendix 2.](https://games.jmir.org/api/download?filename=8266fa56292012c2e7e87d8471dd23c3.docx&alt_name=46358-800079-3-SP.docx) General characteristics of included studies**

| **Author** | **Design** | **Aims** | **Sample Type& Size** | **Data Collection** | **Tests &Analysis** | **Summary of Findings** |
| --- | --- | --- | --- | --- | --- | --- |
| Hu[32] | RCT | To analyze the application effect of VR technology in the PR program for elderly COPD patients complicated with MCI | 60 elderly COPD patients aged 65-80 were randomised into two groups with 30 participants in each group with 23 males, 7 females in the control group and 24 males, and 6 females in the experimental group. | Index of pulmonary function (FEV1%pre, FEV1/FVC), 6MWD, CAT score, and MoCA score were assessed. | Two independent sample t-test and repeated measures to compare data using SPSS 19.0 software. | 1. pulmonary function:   FEV1%pre、FEV1/FVC in the EG after 12 weeks intervention was statistically significantly higher than that in CG.   1. 6MWD (450.30±54.4) in EG was statistically significantly longer than that in CG (416.8±61.7) (P=0.030) at 12 weeks after training; CAT scores(7.4±2.1) in EG was statistically significantly lower than that in CG(10.8±3.6)(P<0.001),while MoCA scores in EG(24.9±2.7) was statistically significantly higher than that in CG(21.0±1.2)(P<0.001)   Virtual reality technology has good application effect in the PR program for elderly COPD patients complicated with MCI and can effectively improve the pulmonary function, cognitive function and exercise tolerance, relieve the dyspnea. |
| LeGear[30] | A within-subjects, randomized cross-over design study | To determine if specific Wii activities resulted in similar energy expenditures to that of a more traditional PR activity | 5 male and 5 female subjects with a mean age of 65 years and a Forced Expiratory Volume in FEV1%pre of 58.1% | Energy expenditure, Heart rate, Sp02, RPE  Borg scale were assessed. | Paired t-tests to compare means | No significant difference was separately identified in total energy expenditure (MD36.3; 95% CI -31.4, 104), heart rate (MD-0.167; 95% CI-4.83, 4.50), RPE (MD 0.100; 95% CI - 0.416,0.616) and Borg scale (MD 0.267; 95% CI -0.00405, 0.537). But significant difference occured in Sp02 (MD2.33%; 95%CI1.52,3.15).  Gaming technology can provide an exercise program that has similar cardiovascular demand to traditional PR programs for patients with COPD. |
| Rutkowski[19] | RCT | To determine 1) whether rehabilitation featuring both VR as well as exercise training provides benefits over ET alone and 2) whether rehabilitation featuring VR training instead of exercise training provides equivalent benefits. | 106 patients with COPD were divided into ETgroup (N=34,18 males and 16 females, mean age,62.1± 2.9),ET+VR group(N=38,19 males, 19 females,mean age,60.6± 4.3),and VR group(N=38,10 males,28 females,mean age 60.4± 4.2） | the Senior Fitness Test (Arm Curl, Chair stand, Back scratch, Chair sit and reach, Up and go), 6MWD were the outcomes | Analysis was performed using linear mixed-effects models. | ET+VR group was superior to ET group in Arm Curl (p<0.003), Chair stand (p<0.008), Back scratch (p<0.002), Chair sit and reach (p<0.001), Up and go (p<0.000), 6MWD (p<0.011). Whereas, the comparison between ET and VR groups showed that VR group was superior to ET group in Arm Curl (p<0.000), Chair stand (p<0.001), 6-min walk test (p<0.031). PR program supplemented with VR training is beneficial intervention to improve physical fitness in patients with COPD. |
| Sutanto[20] | RCT | To evaluate the effectiveness of Wii Fit balance board as an additional tool for exercise training in patients with COPD | This study enrolled 20 participants and 10 patients in experiment group (9 males, 1 female) and 10 male patients in the control group. | 6MWD, TDI, MRC, SGRQ, BODE were assessed | Independent t test, Kruskall---Wallis H test, and X^2^ analysis for dichotomous variables were used | The 6MWD increased from 410.7 (105.3) to 477.5 (122.4) and from 376.6 (81.0) to 420  (77.6) meters, in CG and EG respectively, p = 0.0001 without any difference between groups.  Both groups experienced significant improvements in TDI and SGRQ, but not in MRC and BODE, without any significant difference between groups.  As a result, a Wii Fit balance board based video game program was feasible but did not add any benefit to a well conducted standard exercise training program in patients with COPD. |
| Albores[33] | [pre-post study](http://www.baidu.com/link?url=DZG5yznKJMoZg8pv1w3lwh01O9jaDCWG8a1rtYN0iffjORBHXwhgUS2vh8408j_bYH5uHYaVPDpjFMfS_FFVT4hA7btNLpdV2IjFoKNtdzdw3UAPvjIOo81ur8aQf_07) | To test the effectiveness of a home exercise program based on a user-friendly, computer system, the Nintendo Wii Fit. | 20 clinically stable patients(mean age,68±10) with COPD began a 6-week non-intervention (baseline) period followed by 12 weeks of Wii exercise training at home. | FEV1, the ESWT ,  arm-lift and sit-to-stand repetitions,  the emotion dimension of CRQ were analysed. | Demographic data are presented and a P<0.05 was considered significant.Post hoc analyses by gender and other variables were made using repeated measures analysis of variance. | Baseline data of FEV1 was 45±16%. Following 12 weeks of Wii exercise training, the ESWT scores increased by 131±183 seconds over the baseline determination ( P <0.005), and men had significantly greater increases in the ESWT than women. Significant improvements were also noted in arm-lift and sit-to-stand repetitions, the total score, and the emotion dimension of CRQ. As a result, 12 weeks of regular, home exercise based on an interactive entertainment computer system can lead to positive short-term outcomes. |
| Wardini[34] | [pre-post study](http://www.baidu.com/link?url=DZG5yznKJMoZg8pv1w3lwh01O9jaDCWG8a1rtYN0iffjORBHXwhgUS2vh8408j_bYH5uHYaVPDpjFMfS_FFVT4hA7btNLpdV2IjFoKNtdzdw3UAPvjIOo81ur8aQf_07) | To determine the use of a VGS for exercise training in patients with moderate to very severe COPD undergoing PR | Thirty-two patients were enrolled with a mean age of 66±9 years and a mean FEV1 of 0.72±0.40 L participated. | dyspnea score, FEV1, adherence, oxygen saturation, heart rate, VAS( satisfaction), Borg scale, adverse effects were collected. | Safety, feasibility, enjoyment and adherence were assessed | 25 patients completed this program, and adherence was 76%, with a mean attendance rate of 64±35%. The FEV1 was 0.72±0.40. Dyspnea score was 1.5±1.1 before and 3.2±1.2 after exercise. Mean oxygen saturation changed from 94±3% to 91±5% (P<0.001), while heart rate increased from 88±15 beats/min to 102±18 beats/min (P<0.001); Borg scores statistically increased after intervention. The VAS scores for satisfaction was 8±2.6. Adverse effects were reported including chest pain, low oxygen saturation during exercises.  Moderate exercise using a VGS was safe, feasible and enjoyed as an adjunct to inpatient PR. |
| Parent[35] | [pre-post study](http://www.baidu.com/link?url=DZG5yznKJMoZg8pv1w3lwh01O9jaDCWG8a1rtYN0iffjORBHXwhgUS2vh8408j_bYH5uHYaVPDpjFMfS_FFVT4hA7btNLpdV2IjFoKNtdzdw3UAPvjIOo81ur8aQf_07) | To observe the feasibility of using this device safely and easily with COPD patients | 14 participants (8 men, 69±6 years, 6 women, 74±6 years),with a FEV1 % predicted to be 44±14.8%, for the men it was 37.4±13.5% and for the women 52.8±12.3% performed exercise games | the respiratory parameters (ventilation and breathing rate) and the Borg score were collected. | An ANOVA repeated measures were effectuated using SPSS 20 (IBM, USA) | Average and peak minute ventilation and METs peak were respectively: Stunt Run game (lifting knees on spot) 25.3±6.8, 33.5±8.2 L/min, and 4.2±1.5 METs; Arctic Punch game (punching targets): 23.1±5.6, 31.8±9.8 L/min, and 3.7±1.2 METs; To the Core game (core twist), 22.2±7.3, 29.2±9.9 L/min, and 3.3±1.1 METs; and Squat me to the Moon game (sitting to standing), 27.8±6.7, 36.8±11.1 L/min, and 4.4±1.1 METs.  There was no statistical difference in Borg scores after intervention.  Games could be a good tool in order for COPD patients to exercise at home. |
| Simmich[18] | RCT | To evaluate the feasibility of a co-designed mobile game by examining the usage of the game, subjective measures of game engagement, and adherence to wearing activity tracker | A totol of 18 patients were enrolled and out of 9 of 18 (4 males, 5 females, mean age70±6)were in the experimental group, 9 patients(3 males, 6 females, mean age 65±7) were in the control group. | the usage of the game, subjective measures of game engagement, Intrinsic Motivation Inventory, and adherence to wearing activity trackers, MVPA were recorded. | Data were analyzed and visualized using Python (Python 3.7;Python Software Foundation). | Participants used the game to record physical activity on 58.6% (82/141) of the days the game was available. The highest scores on the Intrinsic Motivation Inventory were seen for the value and usefulness subscale, with a mean of 6.38 (SD 0.6). Adherence to wearing Fitbit was high, with participants in both groups recording steps on >80% of days. Usage of the game was positively correlated with changes in daily steps but not with MVPA.  The co-designed mobile app shows promise as an intervention |
| Mazzoleni[31] | RCT | To evaluate the effectiveness of an interactive videogame system in addition to a supervised PR in patients with chronic respiratory diseases. | A totol of 40 patients were enrolled and out of 20 of 40 were in EG (mean age 68.9±11), and 20 in the CG(mean age, 73.5±9.2). | MRC scale, SGRQ, 6MWD, TDI, BDEI, HRQL, acceptability of PR were assessed. | A Student’s t-test, A one-way Analysis of Variance (ANOVA) were employed using SigmaStat software | Improvement in 6MWD was significantly greater in EG (97.4± 64.8) than in CG(61.1±28.3)(P=0.028).  Dyspnea employed by the MRC improved without statistical difference after intervention between both groups.  Depression employed by BDEI improved without statistical difference after intervention between both groups  Compared to controls(2.2±1), patients in the EG (3.9+1.9) showed a greater improvement in TDI (P<0.001)  HRQL: no significant different was found between EG group and CG group in SGRQ (P=0.657) (10.8±12.4 VS 12.7±14.9, p=0.657).  Acceptability: the two groups showed similar acceptability to the their protocols (42.4±3.5 vs 43.9±3.0; p Z 0.169, in EG and CG respectively).  The addition of an interactive video game training was more effective for improving some parameters of exercise tolerance and dyspnea, although did not result in better psychological status nor it was better accepted than the standard PR in patients with chronic respiratory diseases. |
| Zhou[21] | RCT | To explore the effect of exercise program based on somatosensory interactive games in elderly patients with COPD. | There are 130 COPD patients enrolled in experiment group(48 males, 13 females, mean age: 70.54±6.89) and control group(46 males, 12 females, 71.38±5.78) with 65 patients in each group | 6MWD , FEV1, FEV1/FVC,  Borg scale,  mMRC,  Compliance rate were evaluated. | SPSS 25.0 software was employed to analyze data with Independent t test. | After 18 weeks, the 6MWD of EG was (523.27±29.12)m, and FEV1、FEV1/FVC、mMRC were higher than those of CG, the differences were statistically significant. The dyspnea assessed by Borg scale improved without significant difference. Compliance rate in the EG was 83.61%, which was higher than that of CG, and there was significant difference between two groups.  Somatosensory game interactive training can improve the exercise compliance, physical activity and lung function of elderly patients with COPD in stable period |
| Jin[22] | RCT | To explore the effects of somatosensory interactive games on the balance function of those patients who were in the AECOPD. | There are 66 COPD patients enrolled in EG (27 males, 6 females, mean age:（74.15士75) and CG(27 males, 6 females, mean age:（73.97士10.14) with 33 patients in each group | Brief-EESIbst, days of hospitalization was assessed. | SPSS 19.0 software was employed to analyze data with Independent t test. | After the intervention, the Brief BESTest scores of EG(16.73 士 3.02) was significantly higher than that of the CG(13.88 +4.39 )(P<0.01).  Somatosensory interactive games can improve the balance function of AECOPD patients without prolonging their days of hospitalization from statistical insights between two groups. |

AVGs: interactive training videos, AECOPD: acute exacerbation stage of chronic obstructive pulmonary disease, BODE: exercise capacity index, Brief-EESIbst: brief balance evaluation systems test, CG: control group, CRQ: the Chronic Respiratory Questionnaire, CRQ: Chronic Respiratory Disease Questionnaire, CAT: The COPD Assessment Test, EG: experimental group, ET: exercise training, ESWT: Endurance Shuttle Walk Test, FEV1/FVC: the ratio of the forced expiratory volume in the first one second to the forced vital capacity of the lungs, FEV1%pre: forced expiratory volume in one second predicted, FEV1: forced expiratory volume in the first second, HRQL: health status and health-related quality of life, MD: mean difference, MCI: mild cognitive impairment, MoCA: Montreal Cognitive Assessment, MRC: the Medical Research Council score, MVPA: daily steps and daily moderate-to-vigorous physical activity, RPE: rating of Perceived Exertion, TDI :transitional dyspnea index, PR: pulmonary rehabilitation, SGRQ: the Saint George’s Respiratory Questionnaire, VR: virtual reality, VGS : virtual game system, VAS: visual analogue score, 6MWD: the 6-min walk distance.
